# Evaluation of common findings in brain computerized tomography (CT) scan: A single center study

**DOI:** 10.3934/Neuroscience.2020017

**Published:** 2020-08-13

**Authors:** Daniel Chimuanya Ugwuanyi, Tochukwu Florence Sibeudu, Chidmma Precious Irole, Michael Promise Ogolodom, Chukwudi Thaddeus Nwagbara, Adaobi Maryann Ibekwe, Awajimijan Nathaniel Mbaba

**Affiliations:** 1 Department of Medical Radiography and Radiological Sciences, Nnamdi Azikiwe University, Nnewi Campus, Anambra State, Nigeria; 2 Department of Nursing Sciences, Faculty of Health sciences and Technology, Nnamdi Azikiwe University, Nnewi Campus, Anambra State, Nigeria; 3 Rivers State Hospitals Management Board Port-Harcourt River State Nigeria; 4 Cardiology Unit, Department of Internal Medicine, Nnamdi Azikiwe University Teaching Hospital, Nnewi Nigeria; 5 Department of Radiology, Rivers State University Teaching Hospital, Port Harcourt, Nigeria

**Keywords:** brain computed tomography, common findings, exposure

## Abstract

**Background:**

Computed Tomography (CT) is an invaluable imaging tool in the diagnostic workup of patients presenting with head trauma, stroke, brain tumour and epilepsy. The objective of this study was to document the common intracranial pathologies as revealed by CT in our setting and also determine if the indications for CT scan are justified so that patients are not exposed to radiation unnecessarily.

**Materials and methods:**

This was a cross-sectional study carried out in Hansa Clinic Enugu, Enugu State, Nigeria. Demographic data and brain CT radiological reports with imaging findings and clinical indications for patients referred to this study centre from January, 2017 to January 2019 were retrieved from the CT reports' archives and reviewed retrospectively. Relevant information such age, gender, radiological CT findings and clinical indications were collected using structured proforma.

**Results:**

A total of 300 patients' brain CT radiological reports were included in this study. The mean age of the patients was 41.25 ± 16.5 years with majority been within the age group of 31–40 years 92 (30.67%). Out of 300 cases, normal finding was highest 117 (39%) and the least was intracranial physiological calcification, which is 1 (0.33%). Headache is the most common clinical indication, 53 (17.67%) the least was unsteady Gait, which is 3 (1%). The Chi-square test revealed that there was statistically significance relationship between brain CT findings and clinical indications for the investigations (X^2^ = 285.60, p = 0.002).

**Conclusion:**

The study showed that more males than females undergo brain CT scan with headache being the most common presenting complaint. The majority of findings of the brain CT scans in this study are normal despite, myriads of complaints necessitating the investigations. The study also revealed significant association between clinical indications and CT findings.

## 1. Introduction

The invention of computed tomography (CT) in the 1970's significantly changed radiological imaging and medicine as a whole [1]. CT is essential in the medical imaging armamentarium used in the detection, prevention and screening for disease. It is a widely and readily available imaging modality in the developed world and in Nigeria. CT scanning is increasingly used both in research and in clinical medicine with improved image quality obtained especially with the newer helical scanners [2]–[4].

**Figure 1.**
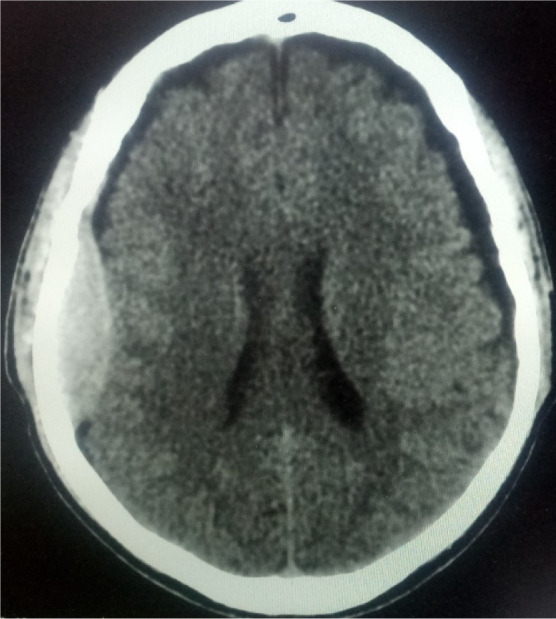
Axial non-contrast brain CT showing acute epidural haemorrhage in the right parietal region.

CT is an invaluable imaging tool in the diagnostic workup of patients presenting with head trauma, stroke, brain tumour and epilepsy. The common brain CT findings vary depending on the indications for the study and may include findings unrelated to the patients' complaints. According to Ijeh-Tarila et al. [5], in a study evaluating stroke patients, the common findings were ischemic infarct, cerebral haemorrhage, cerebral atrophy, cerebral abscess and brain tumour. In another study of patients with head trauma, Ogolodom et al. [6], documented intracerebral hematoma, skull fracture, contusion and extra-axial collection as the common findings. Other common brain CT imaging abnormalities include physiological intracranial calcification, cyst, aneurysm, white matter demyelinating disease, intraventricular haemorrhage, epidural haemorrhage (Figure 1), hydrocephalus, oedema and inflammatory disease. Despite patients' complaints, CT scan may reveal an absence of abnormal findings. Milad and Gamal [7] in a study, which evaluated the profile of head CT findings in 255 patients in their hospital reported 178 cases with no abnormal findings (64%) as the most common outcome.

CT scanners uses high doses of ionizing radiation, which may cause cancer. It is estimated that 0.4% of current cancer in the United States are due to CT scans performed in the past and this may increase to as high as 1.5–2% with 2007 rate of CT usage [8]. Patient exposure is more perilous in CT because, aside using ionizing radiation, the doses are usually higher than for radiographic or fluoroscopic procedures [9]. In addition, the introduction of multi-section CT scanners resulted in relatively large dose increase compared with doses from single-section scanners [10]. It is uncertain whether the cost and risk of brain CT are justified especially when considered vis-à-vis the indications for the study. Furthermore, CT often misses brain dysfunction following mild-to-moderate brain injury [11].

Usage of CT has increased dramatically over the past two decades in many countries [12]. The increased availability of CT scanners and use by especially Nigerian head injury patients has been reported [13]. The advances in technology of the CT scanners available in our environment in recent times have also increased the resolutions and abilities of radiologists in picking up many more subtle findings. To the best of our knowledge, no study has ever examined the common brain CT findings in low-resource setting like ours. The goal of this study was to document the common intracranial pathologies as revealed by CT in our setting and also determine if the indications for CT scan are justified so that patients are not exposed to radiation unnecessarily.

## 2. Materials and methods

This is a cross-sectional study carried out in the Radiology department of Hansa Clinic Enugu, Enugu State, Nigeria after obtaining ethical clearance from the study center. Demographic data and brain CT radiological reports with imaging findings and clinical indications for patients referred to this study center from January, 2017 to January 2019 were retrieved from the CT reports' archives and reviewed retrospectively. Relevant information such age, gender, radiological CT findings and clinical indications were collected using structured proforma. The obtained data were processed on SPSS version 21 and analyzed statistically using both descriptive statistics and inferential statistic (Ch-square test). The level of statistical significance was set at p < 0.05.

## 3. Results

A total of 300 patients' brain CT radiological reports and request forms were evaluated and included in this study. The mean age of the patients was 41.25 ± 16.5 years. Majority (N = 92) within the age of 31–40 years (30.67%), followed by (N = 73) within the age group of 41–50 years (24.33%) and the least (N = 13) were within the age group of <20 years (4.33%) (Table 1). The majority (N = 176) of the cases were males (58.7%), while (N = 124) were females (41.3%) (Table 1). With regards to the common brain CT findings, out of 300 cases, normal finding 39% (N = 117) was highest, followed by Ischemic infarct 20.67% (N = 62) and the least 0.33% (N = 1) was intracranial physiological Calcification. Out of 62 cases of ischemic infarcts, chronic type 61.29% (N = 38) was highest, while acute ischemic infarct was 38.71% (N = 24). Haemorrhage accounted for 14.33% (N = 43) of the total cases studied, with intracerebral haemorrhage 5.33% (N = 16) been highest, followed by (N = 11) subduaral haemorrhage (3.67%), and the least (N = 4) was extradural Haemorrhage (1.33%) (Table 2).

Headache is the most common clinical indication, 17.67% (N = 53), followed by seizure disorders 13.67% (N = 41), loss of consciousness 12.33% (N = 37) cerebrovascular disease (CVD) 11.33% (N = 34), RTA with head injury 9.33% (N = 28) , space occupying lesion, 6.67% (N = 20) and the least was unsteady Gait, which is 1% (N = 3) (Table 3). The Chi-square test revealed that there was statistically significance relationship between brain CT findings and clinical indications for the investigations (X^2^ = 285.60, p = 0.002).

**Table 1.** Frequency and percentage of demographic variables.

	Frequency	Percentage (%)
Age (years)		
≤20 years	13	4.3
21–30 years	37	12.3
31–40 years	92	30.7
41–50 years	73	24.3
51–60 years	59	19.67
≥61 years	26	8.67
Total	300	100.0
Gender		
Male	176	58.7
Female	124	41.3
Total	300	100.0

**Table 2.** Frequency and percentage of common brain CT findings.

CT findings	Frequency	Percent (%)
Cerebral Atrophy	28	9.33
Ischemic Infarct	62	20.67
Normal Brain CT	117	39.00
Fracture	17	5.67
Haemorrhage		
Intracerebral haemorrhage	16	5.33
Intraventricular haemorrhage	6	2.00
Extraduaral haemorrhage	4	1.33
Subdural haemorrhage	11	3.67
Subarahnoid haemorrhage	6	2.00
Tumour	10	3.33
Hydrocephalus	8	2.67
Brain Oedema	4	1.33
Abscess	3	1.00
Metastasis	2	0.67
Meningitis	5	1.67
Intracranial physiological calcification	1	0.33
Total	300	100

**Table 3.** Common Clinical indication for Brain CT.

Clinical Indications	Frequency	Percentage
Loss of Consciousness	37	12.33
Headache	53	17.67
Seizure disorder	41	13.67
Cerebrovascular disease	34	11.33
RTA with Head injury	28	9.33
RVD Encephalopathy	9	3.00
Meningitis	15	5.00
Cerebral Abscess	11	3.67
Intracranial Metastasis	10	3.33
Space occupying lesion	20	6.67
Proptosis	6	2.00
Facial Nerve Palsy	4	1.33
Paranasal Sinusitis	13	4.33
Unsteady Gait	3	1.00
Sinonasal mass	4	1.33
Hyperprollactinemia	5	1.67
Anosmia	7	2.67
Total	300	100

RTA—Road Traffic Accident; RVD—Retroviral Disease.

## 4. Discussion

The demographic description of the cases included in our study revealed that majority of the cases were in their third, fourth, fifth and sixth decades of life with the peak value in the fourth decade of life, which accounted for over 30% of the total study population. Males were highest in number when compared with their female counterparts. This demographic finding is in agreement with the findings of similar studies conducted by Ohaegbulam et al. [14] in a medium-sized city (Enugu, Nigeria) and by Ogolodom et al. [6] in a large city (Port Harcourt, Nigeria). According to Ohaegbulam et al. [14], these categories of people are the most active and productive group in the society and appears to be more prone to both occupational and social risks, while Ogolodom et al. [6] in their study, attributed their finding to the fact that people of this age group commonly involved in the consumption of hard drugs, cultism, militancy activities and disobeying of traffic rules and regulations, especially the male population.

Normal brain CT finding was the most common outcome in our study, despite positive clinical indication to perform the CT scan. This finding is consistent with the findings of similar studies conducted by Haghighi et al. [15] in Taba Radiology centre in Shiraz and Khodapanahandeh and Hadizadeh [16] in Tehran. According to Haghighi et al. [15] finding, out of 167 patients data included in their study, 147 (88.02%) cases were normal findings, while the abnormal findings accounted for only 20 (11.98%) of the total study population. In Khodapanahandeh and Hadizadeh [16] study, CT scans and MRIs were performed for 108 and 11 patients respectively, those with normal findings accounted for 90% of the total case. The differences in the absolute values of the normal findings in our studies could be attributed to the different sample sizes and the nature of the different studies. Contrary to our finding, Ohaegbulam et al. [14] in their study, reported 80.1% of abnormal CT findings with the remaining 19.9% been unremarkable. The nature of our study and that of Ohaegbulam et al., greatly influenced the observed differences in our findings.

In our study, we found that the most common clinical indication for the brain CT investigations was headache. Headache is common complaints in clinical practice, though most of the patients who present with headache have no neurological abnormality on CT and MRI investigations [17]. This finding is consistent with the result of similar study carried out by Haghighi et al. [15]. In a retrospective study conducted by Haghighi et al., which evaluated the abnormal findings in brain CT scans among children in Taba Radiology centre in Shiraz, reported headache as the major complaint for the CT scan, 739 (60.80%). The discrepancies noted in our findings could be ascribed to the different nature and the sample size of the two studies.

It is also important to note that CT often misses mild-to-moderate traumatic brain injury (TBI). CT is widely available and remains a useful tool for visualizing fracture and bleeding, but it contributes little to understanding the pathology of common neurological indications for neuroimaging—acute ischaemic stroke and traumatic brain injury. CT particularly fails in the area of diagnostic imaging of TBI. This is in agreement with the findings of the studies conducted by Haydel et al. [18], Jacobs et al. [19] and Abu-Judeh et al. [20]. Haydel et al. found only 5–10% of CT scans were abnormal in over 4,000 patients with documented mild traumatic brain injury and they concluded that for the investigation of patients with minor head injury, the use of CT can be safely restricted to those who have certain clinical findings. Jacobs et al. documented positive findings of traumatic brain injury by SPECT scan in 126 cases which were negative on CT and MRI. In the study by Abu-Judeh et al., they observed that CT missed or underestimated injury from TBI when compared to the findings from SPECT scans.

In this present study, we found a statistical significant relationship between clinical indications and brain CT findings. This implies that there was justification for the CT requests, although normal findings was found to be highest, which is usually common when the chief complaint is headache as in the case of this study.

## 5. Conclusion

Computed tomography scan is a common imaging modality used to evaluate brain pathologies. The study showed that more males than females undergo brain CT scan with headache being the most common presenting complaint. The majority of findings of brain CT scans in this study is normal despite myriads of complaints necessitating the investigations. The study also revealed significant association between clinical indications and CT findings.
